# Left atrial strain is associated with distinct inflammatory and immune profile in patients with COVID-19 pneumonia

**DOI:** 10.1186/s13089-022-00302-5

**Published:** 2023-01-12

**Authors:** Filipe André Gonzalez, Miguel Ângelo-Dias, Catarina Martins, Rui Gomes, Jacobo Bacariza, Antero Fernandes, Luis Miguel Borrego, EchoCrit Group

**Affiliations:** 1 Intensive Care Department, Hospital Garcia de Orta, Almada, Portugal; 2 NOVA Medical School|Faculdade de Ciências Médicas, NMS|FCM, Universidade Nova de Lisboa, Lisbon, Portugal; 3 Immunoallergy Department, Hospital da Luz Lisboa, Lisbon, Portugal

## Abstract

**Introduction:**

SARS-CoV-2 infection is associated with multiple cardiac manifestations. Left atrial strain (LA-S) by speckle tracking echocardiography (STE) is a novel transthoracic echocardiography (TTE) measure of LA myocardial deformation and diastolic dysfunction, which could lead to early recognition of cardiac injury in severe COVID-19 patients with possible implications on clinical management, organ dysfunction, and mortality. Cardiac injury may occur by direct viral cytopathic effects or virus-driven immune activation, resulting in heart infiltration by inflammatory cells, despite limited and conflicting data are available on myocardial histology.

**Purpose:**

We aimed to explore LA-S and immune profiles in COVID-19 patients admitted to the intensive care unit (ICU) to identify distinctive features in patients with cardiac injury.

**Methods:**

We enrolled 30 patients > 18 years with positive SARS-CoV-2 RT-PCR, admitted to ICU. Acute myocardial infarction and pulmonary embolism were exclusion criteria. On days D1, D3, and D7 after ICU admission, patients performed TTE, hemogram, cardiac (pro-BNP; troponin) and inflammatory biomarkers (ESR; ferritin; IL1β; IL6; CRP; d-dimer; fibrinogen; PCT; adrenomedullin, ADM), and immunophenotyping by flow cytometry.

**Results:**

Patient’s mean age was 60.7 y, with 63% males. Hypertension was the most common risk factor (73%; with 50% of patients under ACEi or ARA), followed by obesity (40%, mean BMI = 31 kg/m^2^). Cardiac dysfunction was detected by STE in 73% of patients: 40% left ventricle (LV) systolic dysfunction, 60% LV diastolic dysfunction, 37% right ventricle systolic dysfunction. Mortality, hospitalization days, remdesivir use, organ dysfunction, cardiac and serum biomarkers were not different between patients with (DYS) and without cardiac dysfunction (nDYS), except for ADM (increased in nDYS group at D7). From the 77 TTE, there was a striking difference between diastolic dysfunction evaluation by classic criteria compared to STE (28.6% vs. 57.1%, *p* = 0.0006). Lower reservoir (*Ɛ*) and contraction (ƐCT) LA-S correlated with IL-6 (*Ɛ*, *p* = 0.009, *r* =  − 0.47; ƐCT, *p* = 0.0002, *r* =  − 0.63) and central memory CD4 T-cells (ƐCT, *p* = 0.049, *r* =  − 0.24). Along all timepoints, DYS patients showed persistent low lymphocyte counts that recovered at D7 in nDYS patients. DYS patients had lower platelets at D3 and showed a slower recovery in platelet counts and CRP levels; the latter significantly decreased at D7 in nDYS patients (*p* = 0.009). Overall, patients recovered with an increasing P/F ratio, though to a lesser extent in DYS patients.

**Discussion:**

Our study shows that LA-S may be a more sensitive marker for diastolic dysfunction in severe COVID-19, which could identify patients at risk for a protracted inflammatory state. A differential immune trait in DYS patients at ICU admission, with persistent lymphopenia, enriched CM T-cells, and higher IL-6 may suggest distinct inflammatory states or migration patterns in patients that develop cardiac injury.

## Introduction

In severe COVID-19 patients, diagnostic transthoracic echocardiography (TTE) allows early recognition of cardiac injury with an impact on clinical management, reducing organ dysfunction and mortality [1, 2]. Notably, the expression of the cardiovascular disease seems to be a marker of a poor prognosis in COVID-19 [3]. More recently, the assessment of left atrial strain (LA-S), assessed by speckle tracking echocardiography (STE), has allowed a more accurate and reproducible analysis of left atrial function [4], correlating more accurately with left ventricle (LV) diastolic dysfunction (LVDD) [5] and with invasive hemodynamics [6]. By measuring less explored diastolic dysfunction parameters, such as LA-S, a broader spectrum of cardiac dysfunction may be recognized, allowing earlier and more effective management in COVID-19 patients.

Here, we aimed to explore diastolic dysfunction parameters and the immune profile in COVID-19 patients admitted to the intensive care unit (ICU) to identify distinctive immune features in patients with cardiac injury.

## Materials and methods

We enrolled 30 patients > 18 years with positive SARS-CoV-2 RT-PCR test who were admitted to ICU between March and September 2022. Acute myocardial infarction and pulmonary embolism were exclusion criteria. On days D1, D3, and D7 after ICU admission, patients performed STE, hemogram, cardiac (pro-BNP; troponin) and inflammatory biomarkers (ESR; ferritin; IL1β; IL6; CRP; d-dimer; fibrinogen; PCT; adrenomedullin, ADM), and immunophenotyping by flow cytometry.

**Fig. 1:**
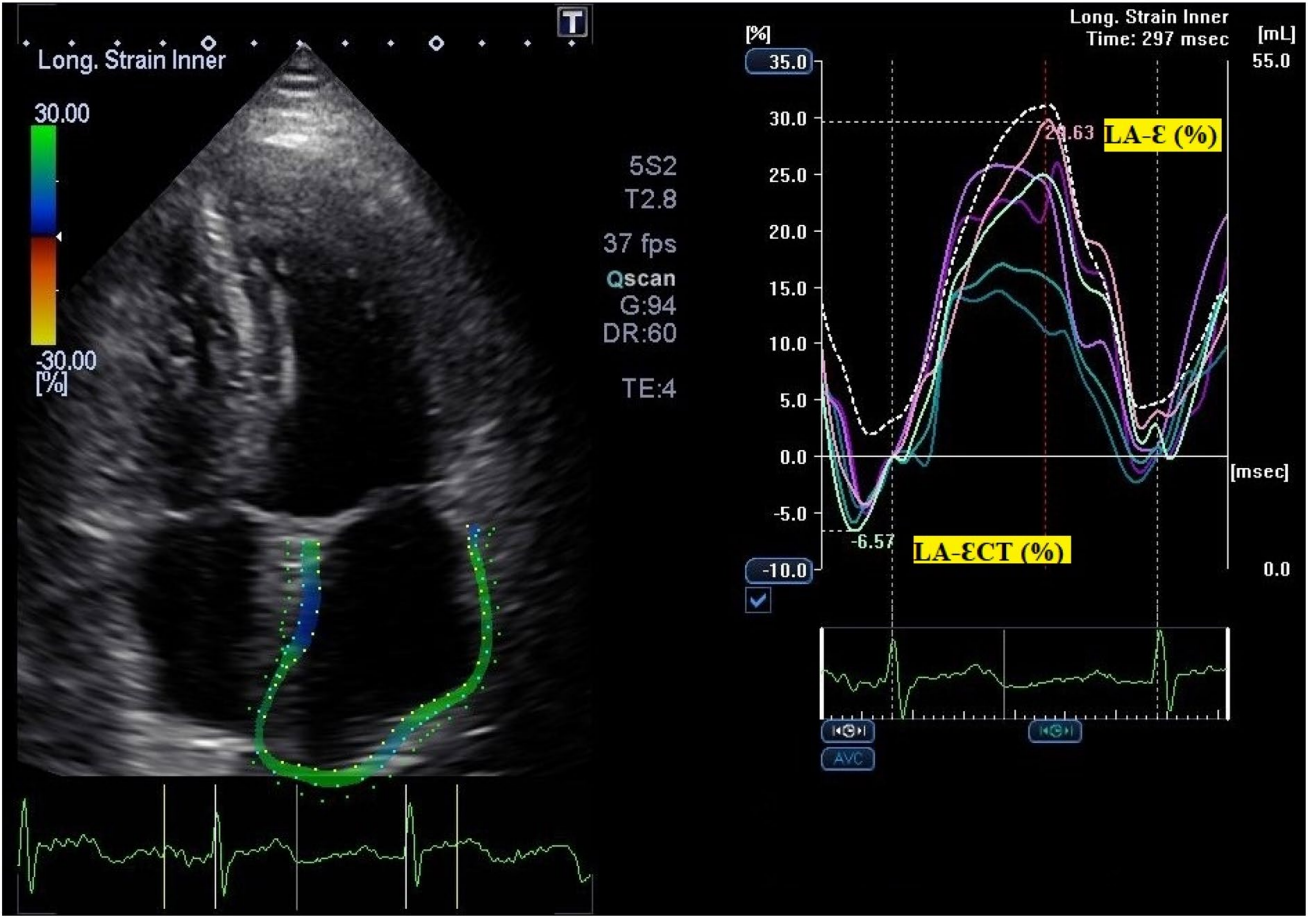
Left atrium strain example with reservoir (*Ɛ*) and contraction (ƐCT) functions highlighted

The presence of cardiac dysfunction (DYS vs. nDYS) was classified according to the European Society of Cardiology (ESC) and American Society of Echocardiography (ASE) guidelines for chamber quantification classification [7]: LV systolic dysfunction (LVSD) was defined as a calculated LVEF < 50% or LVGLS < 20%; RV systolic dysfunction was defined as RV FAC < 35%, TAPSE < 17 mm, S’ < 9.5 cm/s or RVGLS < 20%. LVDD dysfunction was classified by “classic” criteria according to 2016 ASE/EACVI guidelines [8] and by LA-S to assess contractile (ƐCT < 10%) and reservoir (*Ɛ* < 30%) functionality using definitions from previous studies [9] (Fig. 1).

## Results

The patient’s mean age was 60.7 years, with 63% males. Hypertension was the most common risk factor (73%, of which 50% of were under ACEi or ARA), followed by obesity (40%, mean BMI = 31 kg/m^2^). Cardiac dysfunction was detected by STE in 73% of patients: 40% LVSD, 60% LVDD, 37% right ventricle systolic dysfunction. Mortality, hospitalization days, remdesivir use, organ dysfunction, and cardiac and serum biomarkers were not different between patients with and without cardiac dysfunction. The 77 TTE evaluations showed a striking difference between diastolic dysfunction evaluation by classic criteria compared to LA-S (28.6% vs. 57.1%, *p* = 0.0006).

When comparing classic LVDD with LA-S LVDD (Table 1), the latter discriminated longer IVCT (DYS: 70 ms [51–79] vs. nDYS: 50 ms [42–64]) and pre-TE (DYS: 74 ms [67–88) vs. nDYS: 63 ms [42.5–81]), and slightly worse RV systolic function (RV FAC DYS: 41.51% [33.59–48.03] vs. nDYS: 48.61% [40.96–58.32]; RV GLS DYS: 21.85% [18.5–29.24] vs. nDYS: 26.95% [22.38–31.59]) and lower CI (DYS: 2.47 l/min/m^2^ [2.01–2.92] vs. nDYS: 2.83 l/min/m^2^ [2.24–4.11]).

**Table 1:** Comparison of echocardiographic, hemodynamic parameters and cardiac biomarkers between left ventricle diastolic dysfunction by classic criteria vs. left atrium strain

All evaluations median (IQR)	Classic DYS (*n* = 22)	Classic nDYS (*n* = 55)	Classic DYS vs. nDYS*	Strain DYS (*n* = 44)	Strain nDYS (*n* = 33)	Strain DYS vs. nDYS*	Classic DYS vs. strain DYS*
LVEF (%)	55.08 (40.40–63.08)	51.93 (45.02–60.04)	n.s	51.98 (41.40–60.49)	52.67 (47.32–61.64)	n.s	n.s
LV GLS (%)	24.66 (20.85–30.19)	24.06 (20.28–27.80)	n.s	23.72 (18.71–29.00)	25.16 (21.63–28.00)	n.s	n.s
MAPSE (mm)	16.50 (14.70–17.98)	16.20 (15.00–17.80)	n.s	16.05 (14.93–18.25)	16.30 (15.00–17.80)	n.s	n.s
LA-ƐCT (%)	9.66 (6.65–13.23)	11.10 (6.20–15.23)	n.s	6.75 (4.63–9.74)	14.50 (12.69–18.63)	< 0.0001	0.0577
LA-Ɛ (%)	26.35 (21.13–29.07)	30.00 (19.80–38.42)	0.0582	22.15 (17.80–27.30)	37.50 (33.25–42.01)	< 0.0001	0.0403
LV A (cm/s)	78.20 (57.95–90.75)	63.45 (55.78–81.98)	n.s	66.35 (55.40–82.25)	64.20 (57.55–93.65)	n.s	n.s
LV E (cm/s)	59.00 (53.75–78.30)	73.10 (63.20–84.60)	0.0041	67.85 (57.45–79.65)	71.50 (60.25–83.30)	n.s	n.s
LV E/A	0.74 (0.62–1.08)	1.08 (0.89–1.33)	0.0005	0.97 (0.76–1.36)	1.03 (0.82–1.29)	n.s	0.0288
LV e' lat (cm/s)	9.65 (7.15–10.78)	10.80 (9.20–12.80)	0.0114	10.45 (8.13–12.80)	9.80 (9.00–11.45)	n.s	n.s
LV e' sep (cm/s)	6.60 (5.80–8.10)	8.50 (7.00–10.10)	0.0087	7.78 (6.50–11.50)	7.90 (6.50–9.20)	n.s	n.s
LV E/e'	7.62 (5.91–9.39)	7.63 (6.72–9.15)	n.s	7.58 (6.24–9.27)	7.80 (6.81–9.20)	n.s	n.s
LV IVCT (msec)	67.00 (51.00–79.00)	85.00 (71.00–103.50)	n.s	70.00 (51.00–79.00)	50.00 (42.00–64.00)	0.0018	n.s
LV pre-ET (msec)	71.50 (56.00–81.25)	71.00 (54.00–88.00)	n.s	74.00 (67.00–88.00)	63.00 (42.50–81.00)	0.0297	n.s
RV FAC (%)	42.34 (31.21–51.15)	43.03 (40.13–52.28)	n.s	41.51 (33.59–48.03)	48.61 (40.96–58.32)	0.0022	n.s
RV GLS (%)	25.99 (19.11–34.27)	24.03 (18.89–29.22)	n.s	21.85 (18.50–29.24)	26.95 (22.38–31.59)	0.0303	n.s
TAPSE (mm)	21.10 (18.88–23.35)	22.30 (20.50–25.00)	n.s	21.55 (19.45–23.35)	22.80 (20.60–25.10)	n.s	n.s
RV S' (cm/s)	14.90 (13.25–16.28)	15.90 (12.40–18.00)	n.s	14.90 (12.65–17.45)	15.80 (12.50–17.60)	n.s	n.s
RV e' (cm/s)	9.40 (6.68–13.43)	10.20 (8.00–14.40)	n.s	11.25 (8.70–14.98)	9.50 (7.65–12.70)	0.0331	n.s
CI (l/min/m^2^)	2.47 (1.99–3.42)	2.67 (2.20–3.04)	n.s	2.47 (2.01–2.92)	2.83 (2.24–4.11)	0.0473	n.s
NTproBNP (pg/ml)	275.00 (95.30–643.80)	214.00 (84.00–923.00)	n.s	229.50 (79.05–1016.00)	262.00 (107.00–650.00)	n.s	n.s
Troponin (ng/l)	13.00 (13.00–15.17)	13.00 (13–23.70)	n.s	13.00 (13.00–16.55)	13.10 (13.00–20.14)	n.s	n.s

DYS, cardiac dysfunction; nDYS, no cardiac dysfunction; LV, Left ventricle; LVEF, LV ejection fraction; LV GLS, LV global longitudinal strain; MAPSE, Mitral annular plane systolic excursion; LA, Left atrium; LA ƐCT, LA strain contraction function; LA Ɛ, LA strain reservoir function; LV A, LV Atrial contraction wave; LV E, LV Early diastolic filling wave; LV E/A, LV E wave to A wave ratio; LV e' lat, LV tissue doppler lateral e wave; LV e' sep, LV tissue doppler septal e wave; LV E/'e', LV E wave to tissue doppler e wave ratio; LV IVCT, LV isovolumetric contraction time; LV pre-ET, LV pre-ejection time; RV, Right ventricle; RV FAC, RV Fractional area change; RV GLS, RV Global longitudinal strain; TAPSE, Tricuspid annular plane systolic excursion; RV S', RV tissue doppler S wave; RV e', RV tissue doppler lateral e wave; CI, Cardiac index; NTproBNP, NT terminal of prohormone brain natriuretic peptide

*Mann–Whitney test

## Discussion

Our data regarding cardiac dysfunction prevalence in COVID-19 patients is in line with recent evidence, suggesting that 70% of patients with COVID-19 harboured a cardiac injury within the first ICU admission, identified by multimodal cardiac assessment [10]. This incidence is higher than other previously reported values that ranged from 12 to 30% [11–13]. The higher incidence reported by Doyen and collaborators [10] could be explained by either the longitudinal cardiac assessment or the use of a much more sensitive tool to detect LV diastolic dysfunction, as is the STE [5], being simultaneously in line with incidences reported in critically ill patients with sepsis not related to COVID-19 [14]. Thus, it confirms that COVID-19 patients experienced more LV diastolic than systolic dysfunction [15].

Diastolic dysfunction is an elusive pathological condition due to the late definition of its diagnostic criteria, and these strict classical parameters are difficult to apply to critically ill patients, compromising its classification. Recently, some studies have tried to overcome this issue by simplifying and validating specific criteria. Clancy et al. [16] applied the 2016 America Society of Echocardiography (ASE)/European Association of Cardiovascular Imaging (EACVI) guidelines, comparing with the previous 2009 ASE guidelines, achieving 60% of diastolic dysfunction on the first day, with a further 23% having an indeterminate diastolic function, where only 21% had confirmed diastolic dysfunction with 74% having indeterminate diastolic dysfunction, respectively. In addition, Lanspa et al. [17] proposed a simplified definition using only e′ and E/e′, categorizing 87% of patients, compared with 35% of patients using ASE 2009 guidelines. Both groups had similar clinical outcomes. Nevertheless, the massive diffusion of echocardiography provides a more precise appreciation of its burden in critically ill patients. Recently, Filippo Sanfilippo et al. reviewed and explained extensively the challenges of diagnosing diastolic dysfunction in critically ill patients [18].

In this context, a systematic echocardiographic evaluation of 100 COVID-19 patients by Szekely et al. revealed a LVDD as high as 90% in their cohort with a mean age of 66 years, despite a preserved LV ejection fraction [19]. Alongside the subclinical ventricular relaxation impairment (given the advanced age and co-morbidities such as systemic hypertension), the conglomeration of factors specific to COVID-19 such as systemic inflammatory milieu, endothelial dysfunction, microvascular thrombosis, arrhythmias, disturbed ventricular cross-talk (owing to the concomitant right ventricular dysfunction resulting from pulmonary hypertension), and myocardial oxygen supply–demand perturbations, can contribute significantly to the LVDD with a subsequent accentuated potential to culminate as heart failure with preserved ejection fraction (HFpEF) [15].

Few studies have addressed the evolution of the immune profile in COVID-19 patients with cardiac injury. Namely, Laing et al. [20] accomplished an exhaustive immunological analysis, where they identified discrete changes in the compartments of circulating B and myelomonocytic cells, along with profoundly altered T cell phenotypes, upregulation of several cytokines/chemokines and SARS-CoV-2-specific antibodies. Moreover, another group studied the association between the immune profile and cardiac injury in COVID-19 patients, suggesting that the numbers of T and B lymphocytes were significantly decreased in the group with cardiac injury [21].

Our study shows that LA-S may be a more sensitive marker for diastolic dysfunction in severe COVID-19, identifying patients at risk for a protracted inflammatory state.

In fact, we have previously described that a differential immune trait is present in patients at ICU admission, with persistent lymphopenia, enriched central memory CD4 T cells, and higher serum levels of IL-6, suggesting a distinct inflammatory state and migration patterns in patients that develop cardiac injury [22]. Thus, an improved comprehension of the likelihood of an altered diastology in COVID-19 patients is doubtlessly pivotal in staging a more well-directed management approach, wherein targeted echocardiographic surveillance, cardiac and immune-inflammatory biomarkers, combined heart–lung ultrasound and inodilators, can assist the overall management of this critically ill cohort.

Within our limitations, it is worth mentioning the small sample size that could have underpowered our study for more clinically significant outcomes such as mortality, mechanical ventilation, shock, ICU and hospital length of stay. Nevertheless, the repeated measures along the time course may have partially overcome this issue for the other results.

## Conclusions

Our study shows that LA-S may be a more sensitive marker for diastolic dysfunction in severe COVID-19, particularly patients at ICU admission with persistent lymphopenia, enriched CM T-cells, and higher IL-6, which may suggest a differential immune trait in cardiac injury COVID-19 patients in ICU.

## Data Availability

The data sets used and/or analyzed during the current study are available from the corresponding author on request.
